# Quantitative Analysis of Deer Bone Hydroethanolic Extract Using Label-Free Proteomics: Investigating Its Safety and Promoting Effect on Mouse Embryonic Osteoblastic Progenitor Cell Proliferation

**DOI:** 10.3390/nu16223807

**Published:** 2024-11-06

**Authors:** Yanlu Li, Junxia Ma, Yingshan Jiang, Yanchao Xing, Zhongmei He, Weijia Chen, Yan Zhao, Jianan Geng, Ying Zong, Rui Du

**Affiliations:** 1 College of Chinese Medicinal Material, Jilin Agricultural University, Changchun 130118, China; l1615564677@163.com (Y.L.); jxiama@163.com (J.M.); 15754307745@163.com (Y.J.); 18345780732@163.com (Y.X.); heather78@126.com (Z.H.); chenweijia_jlau@163.com (W.C.); zhyjlu79@163.com (Y.Z.); gengjianan@jlau.edu.cn (J.G.); 2 Laboratory of Production and Product Application of Sika Deer, Jilin Agricultural University, Changchun 130118, China; 3 Key Lab of Animal Production, Product Quality and Security, Ministry of Education, Jilin Agricultural University, Changchun 130118, China

**Keywords:** deer bone, multilevel mass spectrometry, cytotoxicity, acute toxicity, subacute toxicity

## Abstract

Background: Deer bone is rich in proteins and free amino acids, offering high nutritional value and benefits such as strengthening bones and antioxidant properties. However, the development and utilization of deer bone resources are limited, and the safety evaluation of health foods is incomplete. Methods: We established a hydrogen ethanol extraction method for deer bone and analyzed the components of the deer bone hydroethanolic extract (DBHE) using liquid chromatography–tandem mass spectrometry (LC-MS/MS), gas chromatography–mass spectrometry (GC-MS), and inductively coupled plasma mass spectrometry (ICP-MS). Results: Using Label-free proteomics technology, we identified 69 proteins and 181 peptides. We also quantified 16 amino acids, 22 fatty acids, and 17 inorganic elements. Finally, we evaluated the safety of DBHE both in vitro and in vivo. The results indicated that DBHE did not exhibit any toxic effects at the doses we tested and can promote the proliferation of mouse embryonic osteoblastic progenitor cells (MC3T3-E1), demonstrating potential efficacy against osteoporosis and arthritis. Conclusions: This study provides a theoretical basis for the quality control, processing, and resource development of deer bone.

## 1. Introduction

Deer bones, from those of deer species being raised on a large scale in China, New Zealand, and other countries, such as the Sika deer and red deer, are a valuable resource with enormous nutritional and medicinal potential [1]. These bones are rich in protein, free amino acids, chondroitin sulfate, organic calcium, and phosphate ions, which play a crucial role in promoting bone health, enhancing immunity, and exhibiting anti-inflammatory, antioxidant, and anti-aging properties [2,3,4,5]. Despite the growing interest in utilizing deer bones, their full potential remains largely untapped due to limited research and development efforts. At the same time, the incomplete safety evaluation of deer bone-related products also hinders the development and utilization of deer bone functional foods.

Due to poor digestion and absorption, direct consumption of bone-based foods might limit the effective utilization of these active components. Consequently, the safe and effective extraction of bone components has become crucial for developing their nutritional and medicinal value. There have been studies using infrared radiation, ultrasound, and fermentation to remove the fat and protein components from bones, as well as using autoclaving to soften the bones, resulting in deer bone products [6,7]. Further in-depth research indicated that orally administered fermented deer bone collagen peptides (FCP), obtained through fermentation with *Lactobacillus acidophilus*, can enhance skin hydration and antioxidant capacity in d-galactose-induced aging mice and regulate the synthesis and degradation of collagen [8]. At the same time, studies have evaluated the bioavailability of yak bone collagen hydrolysate (YBCH) and screened out seven typical bioactive peptides [9]. These provide us with a very important reminder that the key factor affecting substance activity is not only the extraction method but also the choice of extraction solvent. In addition to the traditional water extraction method [10], relevant studies have reported the antioxidant effects of deer antler wine [11]. However, no novel solvent for deer bone extraction has been proposed. Therefore, a hydroethanolic method was chosen to extract deer bone, and the composition of the deer bone hydroethanolic extract (DBHE) was systematically identified using multilevel mass spectrometry. This provides a theoretical foundation for the development of functional foods and health products derived from deer bone.

The current study on deer-derived items primarily centers around antlers [12], with only a few studies investigating deer bone. This lack of research limits the progress and utilization of deer bone to some degree. Although interest and usage of deer bone have increased, our understanding of the safety of deer bone extract remains limited, hindering the development of deer bone-related health products [13]. According to the records and usage information of deer bones in traditional Chinese books, we preliminarily conclude that they are relatively safe. However, comprehensive toxicological studies are still required to fully assess their safety [14]. Therefore, in this study, we conducted a comprehensive safety assessment of DBHE both in vitro and in vivo. Based on the significant osteogenic role of deer bone in traditional Chinese medicine, we selected MC3T3-E1 cells to evaluate the effects of DBHE on cell proliferation and apoptosis [15]. Additionally, the safety of DBHE was assessed at the animal level from both acute and subacute perspectives [16], laying a solid theoretical foundation for the development of more health-promoting deer bone products.

## 2. Material and Methods

### 2.1. Materials, Animals, and Instruments

The Sika deer bone samples were purchased from Haixia Deer Product Distribution Office in Shuangyang District, Jilin Province. Mouse embryonic osteoblast precursor cells (STCC20026P) were purchased from Wuhan Sevier Biotechnology Co., Ltd. (Wuhan, China). The Annexin V-FITC apoptosis detection kit (BB-4101) was supplied by BestBio Co., Ltd. (Shanghai, China). Cell Counting Kit-8 (BS350B) was purchased from Lanjieke Technology Co., Ltd. (Beijing, China). Hematoxylin (BA-4097) and eosin (BA-4022) were purchased from Zhuhai Beso Biotechnology Co. (Zhuhai, China). LC-MS-grade methanol (MeOH) was purchased from Fisher Scientific (Loughborough, UK). 2-Amino-3-(2-chloro-phenyl)-propionic acid was obtained from Aladdin (Shanghai, China). Anhydrous ethanol, xylene, and neutral glue were purchased from Sinopharm Chemical Reagent Co. (Shanghai, China). The biochemical quality control products, total protein (TP), alanine amino transferase (ALT), aspartate transaminase (AST), albumin (ALB), creatinine (CREA), UREA, triglyceride (TG), total cholesterol (TC), glucose (GLU), and calcium (Ca) were purchased from Shenzhen Myriad Biomedical Electronics Co. (Shenzhen, China). Fe was purchased from Beckman Coulter Diagnostics (Brea, CA, USA).

Healthy Wistar rats (4 to 8 weeks, 180 to 220 g, SPF) were bought under license number SCXK (JI) 2023-0002 from Yisi Experimental Animal Technology Corporation (Changchun, China). All animal experiments have been approved by Jilin Agricultural University’s Animal Welfare and Ethics Committee (Ethical Review Acceptance No: 20211011003). Male and female rats were placed in separate cages made of sterile polypropylene. All the animals were fed standard basic feed and cold boiled water. All animals were acclimatized to their new environment for one week before the start of this study. The animals were kept in a normal (25 ± 2 °C) environment with 12:12 h light–dark cycle. The trials were conducted in compliance with National Institutes of Health Guidelines on Laboratory Animals [17].

Automatic amino acid analyzer (L-8900), Hitachi, Japan. Liquid chromatography (Easy nLC/Ultimate 3000, C18 1.9 μm 150 μm × 120 mm), Thermo Fisher Scientific, Waltham, MA, USA. Gas chromatograph (7980B), Agilent Technologies, Santa Clara, CA, USA. Orbitrap Fusion Lumos mass spectrometer and mass spectrometric column (C18 1.9 μm 150 μm × 120 mm, Ion Source Type is NSI), Thermo Fisher Scientific. Inductively Coupled Plasma Mass Spectrometer Nexlon 1000 G, PerkinElmer, Hopkinton, MA, USA. BD FACSVerse™ Cell Analyzer, Becton, Dickinson and Company, Franklin Lake, NJ, USA. Fully Auto Hematology Analyzer (PE-6800), Shenzhen Prokan Electronics Co., Ltd. (Shenzhen, China). Veterinary biochemistry analyzer (BS-240VET) produced by Shenzhen Myriad Biomedical Electronics Co., Ltd. (Shenzhen, China). Paraffin embedding machine (Leica EG1140) and paraffin sectioning machine (Leica RM2255), Zhejiang Jinhua Kedi Instrument Equipment Co., Ltd. (Zhejiang, China). Electronic Balance (FA1004N) Shanghai Precision Scientific Instruments Co., Ltd. (Shanghai, China) Optical microscope (OlympusBX51), Olympus, Nagano, Japan. Image Analysis System (NIS-ELEMNT BR) from NIKON, Yokohama, Japan.

### 2.2. Sample Preparation

Fresh Sika deer leg bones were crushed into pieces measuring approximately 2–3 cm in size. The residual tendon, skin, bone marrow, and a small amount of muscle were meticulously removed from the bones. Following cleaning with deionized water, the bones were soaked in petroleum ether for three consecutive days to remove the fat. The deer bones were treated with 4% hydrochloric acid for 24 h to undergo demineralization. Upon completion of the decalcification process, the bones were thoroughly washed several times with deionized water, and the pH value was adjusted back to neutrality. A 65% ethanol solution was added to the bones at a solid-to-liquid ratio of 1:8 (g:mL). Reflux extraction was carried out at 65 °C. The extraction procedure was repeated three times, with each extraction lasting three hours. The extracts from each cycle were combined and then centrifuged at 8000 rpm for 15 min. The supernatant was lyophilized, resulting in a light-yellow powder that was designated as DBHE (Deer Bone Hydroethanolic Extract) and stored at 4 °C until use.

### 2.3. Measurement of Protein Composition by Label-Free Proteomics

#### 2.3.1. Protein Extraction and Quantitative Quality Inspection

For sample lysis and protein extraction, an SDT buffer (4% SDS, 100 mM Tris-HCl, 1 mM DTT, pH 7.6) was employed. Protein quantification was conducted using a BCA protein assay kit (Bio-Rad, Hercules, CA, USA). Proteins larger than 100 kDa were resolved on a 5% Tris-HCl polyacrylamide gel (Criterion Ready Gel, Bio-Rad), with 20 μg of protein loaded per well. Electrophoresis was performed at a constant current of 5 mA/gel for 16.5 h. The running buffer contained 25 mM Tris, 192 mM glycine, and 0.1% SDS, suitable for both the upper and lower chambers of the Criterion Cell system (Bio-Rad). Molecular weight markers were loaded at 10 μL per well, including Bio-Rad’s pre-stained Kaleidoscope Standard and HiMark ™ Pre-stained Standard (Invitrogen, Waltham, MA, USA), to help identify the protein bands. Coomassie Blue (consisting of 17 percent ammonium sulphate, 2 percent phosphoric acid, 34 percent methanol, and 0.04 percent Coomassie Brilliant Blue G-250) was applied to the gels at room temperature for 48 h.

#### 2.3.2. Protein Digestion

After quantifying the protein, 30 μg of protein was subjected to treatment with 10 mM DTT at a temperature of 37 °C for a duration of 1 h. This was followed by alkylation using 50 mM IAA in a dark environment at room temperature for an additional hour. After a 10 min burst with 5 mM DTT, proteins were ultrafiltered using an Amicon Ultra-0.5 centrifugal filter (UFC503096, Millipore, Burlington, MA, USA). Subsequently, they were washed twice with 100 μL of 8 M urea and three times with 100 μL of 50 mM NH_4_HCO_3_. The proteins, in a 50:1 ratio of protein and trypsin, were enzymatically digested at 37 °C for 18 h. The peptides obtained were purified, dried, and then dissolved in a solution containing 0.1% formic acid (FA) in a volume of 40 μL. The concentration of the peptides was determined by measuring the absorbance at a wavelength of 280 nm (OD_280_).

#### 2.3.3. LC-MS/MS Analysis

A high-performance liquid chromatography (HPLC) system was employed to separate the DBHE samples using two buffers: buffer A, consisting of 0.1% formic acid, and buffer B, comprising 80% acetonitrile and 0.08% formic acid. Prior to commencing the run, the liquid chromatography column was balanced with a 100% solution of buffer A. The liquid chromatography elution gradient starts at 0 min with 95% A (0.1% FA in H_2_O) and 5% B (0.08% FA in 80% ACN, H_2_O) at 600 nL/min. At 8 min, the composition is 90% A and 10% B. By 58 min, it changes to 76% A and 24% B. At 70 min, it shifts to 68% A and 32% B. From 71 to 78 min, the composition is 5% A and 95% B, all with a constant flow rate of 600 nL/min. The samples were autonomously introduced into an MS column for analysis using the Orbitrap Fusion Lumos instrument manufactured by Thermo Scientific. The MS settings include a source voltage of 4 kV, a source temperature of 350 °C, a desolvation gas flow rate of 10 L/min, and a cone gas flow rate of 50 L/hr. Additionally, the collision energy was set to 30 eV, and the scan time was 0.2 s. The mass spectrometer was operated in positive ion mode with a mass range of *m*/*z* 100–1500. The parameters are determined in the following manner: The analysis duration is 78 min. The detection mode is configured for positive ions. The scanning range is from 300 to 1400 *m*/*z*. The primary mass spectrometry resolution is set to 120,000. The AGC target is 5 × 10^5^. The maximum injection time (IT) is 50 milliseconds. The duration of dynamic exclusion was configured to be 20 s. The MS2 activation method employed is HCD (Higher-energy Collisional Dissociation). The isolation window has been decreased to 1.6 *m*/*z*. The micro-scan is configured to perform a single scan. The second maximal injection time (IT) is set to 35 ms. Lastly, the normalized collision energy is adjusted to 33 eV.

#### 2.3.4. Database Search and Bioinformatical Analysis

Protein and peptide identification was achieved through a searchable database. The database utilized for this purpose was the Canadian deer protein database, which was downloaded from NCBI [Cervus_canadensis_GCF_019320065.1_protein.faa]. The software employed for searching was MaxQuant version 2.1.4.0 [18,19,20]. Gene Ontology and KEGG pathway enrichment analysis was conducted using the EBI database and InterProScan software version 5.31–70.0.

### 2.4. Measurement of Amino Acid Content

A 0.02 g DBHE was accurately weighed, followed by the addition of 8 mL 50% HCl. Nitrogen was then flushed into the vessel to create a nitrogen-filled environment. The sample was placed in an oven maintained at 110 °C for a digestion period of 24 h. After completion of hydrolysis, wait to return to room temperature, followed by filtration and addition of water to 50 mL through a volumetric flask. Subsequently, 0.5 mL of the filtrate was transferred into a clean centrifuge tube for further purification. The solvent was removed using a vacuum centrifugal concentrator set at 60 °C. Repeat the above evaporation operation 1–2 times to ensure integrity. A 1 mL aliquot of water was added to reconstitute the concentrate, which was then shaken vigorously to ensure thorough mixing. The solution was passed through a 0.22 μm filter membrane to remove any particulates. The filtered solution was transferred to a clean vial and prepared for analysis using an automatic amino acid analyzer (Hitachi, Tokyo, Japan, model L-8900).

### 2.5. GC-MS Profiling of Fatty Acids

Firstly, 5.9 mg of DBHE lyophilized powder was taken out as a sample. The samples were placed in 2 mL EP tubes, and 500 μL of extraction solution (isopropanol/*n*-hexane = 2:3, 0.2 mg/L internal standard) was added. Following vortexing for 30 s, the sample underwent ball mill homogenization at a frequency of 40 Hz for a duration of 4 min, followed by ultrasonic treatment in ice water for 5 min. Centrifugation at 12,000 rpm for 15 min at 4 °C. The extraction process was repeated once. The supernatant liquid was mixed together and agitated, and 800 μL was evaporated using nitrogen gas. A solution of methanol and trimethylsilyl diazomethane at a ratio of 1:2 and a volume of 500 μL was added. The mixture was allowed to react for 30 min at room temperature and then dried once more. Subsequently, 160 μL of *n*-hexane was added to the tube. The tube was then centrifuged at 12,000 rpm for 1 min. The resulting supernatant was then made ready for GC-MS.

The GC-MS parameters utilized were as follows: a sample volume of 1 µL was injected in split mode (5:1) with a front inlet septum purge flow of 3 mL/min. Helium served as the carrier gas, operating at a column pressure of 46 psi through a DB-FastFAME column (90 m × 250 µm × 0.25 µm). The oven temperature ramp began at 50 °C for 1 min, followed by an increase at 50 °C/min up to 200 °C, which was held for 15 min. The temperature was then increased at 2 °C/min to reach 210 °C, held for another minute, and finally increased at 10 °C/min to 230 °C, where it was held for 15 min. The front injection temperature was set to 240 °C, with the transfer line and ion source temperatures also at 240 °C and 230 °C, respectively. The quadrupole temperature was maintained at 150 °C, and an electron energy of −70 eV was applied. The mass range was set from *m*/*z* 33 to 400, operating in both scan and SIM modes, with a solvent delay of 7 min.

### 2.6. ICP-MS Detection of Inorganic Elements

Weigh 0.1 g of DBHE freeze-dried powder into a PTFE-lined microwave digestion vessel, add a small amount of distilled water to moisten the sample, and then add 5 mL of HNO_3_ and let it stand for 1 h. Afterward, 1 mL of H_2_O_2_ was added, and the mixture was homogenized, sealed, and placed into the microwave digester to proceed with the digestion according to the digestion program. The digested sample was then placed on an acid-evaporation instrument for acid removal until the liquid volume was reduced to the size of a soybean. The remaining liquid was diluted to a final volume of 50 mL with distilled water. The diluted sample was filtered through a 0.22 μm membrane to obtain the test solution, which was then analyzed using ICP-MS [21].

The ICP-MS instrument conditions were set as follows: RF power of 1550 W, plasma flow rate of 15 L/min, nebulizer flow rate of 0.88 L/min, carrier gas flow rate of 1.05 L/min, collision cell gas flow rate of 4.0 mL/min, sample depth set to 8.0 mm, and scanning method set to peak jumping, with 3 repetitions per measurement and a solution stabilization time of 45 s. Sensitivity parameters were configured to ensure Be > 5000, In > 80,000, and U > 6000 counts, while the oxide ratio was maintained below 2.5% (CeO/Ce), and the double charge ratio was maintained below 3% (Ce^2+^/Ce), ensuring optimal performance and accurate analysis with the NEXION 1000G ICP-MS instrument, PerkinElmer Inc., Waltham, MA, USA.

### 2.7. Cytotoxicity Assay

#### 2.7.1. Cell Counting Kit-8 Test Cell Vitality

MC3T3-E1 cells in logarithmic growth phase were distributed onto 96-well plates at a density of 1 × 10^4^ cells per well. The plates were then placed in a 5% CO_2_ environment at a temperature of 37 °C and incubated for 24 h. Subsequently, the concentrations of DBHE (15.625, 31.25, 62.5, 125, 250, 500, 1000 μg/mL) were delivered individually at their respective concentrations. Following a 24 h treatment period, 10 μL of CCK-8 was introduced to each well, and the plates were returned to the incubator for an additional 2 h of incubation in the absence of light. Ultimately, the microplate reader was utilized to measure the absorbance at 450 nm in order to evaluate the viability of the cells [22].

#### 2.7.2. FITC-Labeled Annexin V Apoptosis Assay

MC3T3-E1 cells were placed in 6-well plates with a density of 5 × 10^5^ cells per well and left to incubate for 24 h in a CO_2_ incubator. The control group was provided with a new medium, whereas the remaining groups were administered with DBHE at concentrations of 250, 500, and 1000 μg/mL, respectively [23]. Subsequently, all groups were incubated in a CO_2_ incubator for a duration of 24 h. Following incubation with the designated treatments, the cells were isolated without the use of EDTA trypsin, subjected to centrifugation for a duration of 5 min at a force of 100× *g*, and the supernatant was meticulously extracted. The cells were rinsed in chilled PBS and then subjected to centrifugation again. A suspension of 5 × 10^6^ cells/mL was combined with Annexin V/FITC and PI and then incubated with FlowJo-V10.4.

### 2.8. In Vivo Toxicity Study

#### 2.8.1. Acute Toxicity Study

The evaluation of acute toxicity was conducted in accordance with the established protocol [24]. Forty healthy Wistar rats were randomly divided into four groups of ten rats each (five males and five females). Post-administration of DBHE at doses of 0, 2000, 4000, and 8000 mg/kg, the animals were closely observed for 4 h. Thereafter, for a period of 14 days, all mortalities and changes in general behavior were meticulously documented. Daily weight measurements, along with food and water consumption, were recorded to monitor for any alterations. To determine the daily intake of food and water for each rat, a fixed amount of food and water was provided, and the remaining quantities were measured 24 h later. The difference between the initial provision and the subsequent remainder was recorded as the daily food and water intake per rat. Upon completion of the treatment phase, all animals were humanely euthanized using chloral hydrate anesthesia. Major organs, including the liver, heart, spleen, kidneys, lungs, testes, and ovaries, underwent visual inspection. Their weights were recorded, and organ coefficients were calculated.

#### 2.8.2. Four-Week Subacute Toxicity Study

For the subacute toxicity investigation, a total of forty Wistar rats were used. The rats were between 4 and 8 weeks old and weighed between 180 and 220 g. They were of SPF grade. The rats were randomly divided into four groups, with each group consisting of ten rats (five males and five females). The animals were administered doses of 0, 2000, 4000, and 8000 mg/kg DBHE per day for 28 consecutive days, with access to food and water provided after administration. Throughout this 28-day duration, the rats were closely monitored for any changes in their appearance and physical condition, as well as being observed for changes in the skin, mouth, nose, behavior, respiration, glandular secretions, feces, and urine. Daily morning checks were conducted to assess mortality and behavioral alterations. Furthermore, daily recordings were made of the rats’ body weights and their consumption of water and food to maintain a comprehensive record of their physiological status.

Following the four-week subacute toxicity test, blood samples were taken from the posterior orbital venous plexus for biochemical and hematological analysis. An automated blood cell analyzer was used to analyze the hematology of white blood cells (WBC), red blood cells (RBC), lymphocytes (LYM), hemoglobin (HGB), and platelets (PLT) [25]. Indicators of liver and kidney function (TP, ALT, AST, ALB, UREA, CREA), glycolipid metabolism (GLU, TG, TC), and elemental content (Ca, Fe) were detected in rat serum [26]. Finally, the animals were euthanized, and the heart, liver, spleen, lung, kidney, ovary, testis, and knee joint of the rats were removed for calculation of organ index and performed histopathological examination.

### 2.9. Statistical Analysis

The experimental data were reported in terms of the mean value plus or minus the standard deviation (SD). Statistically significant differences, with a significance level of *p* < 0.05, were evaluated using one-way Analysis of Variance (ANOVA). The analyses in the study were performed using Origin 8.5 software (Origin Lab Corporation, Northampton, MA, USA).

## 3. Results

### 3.1. Protein Identification and Quantitative Results

**Figure 1:**
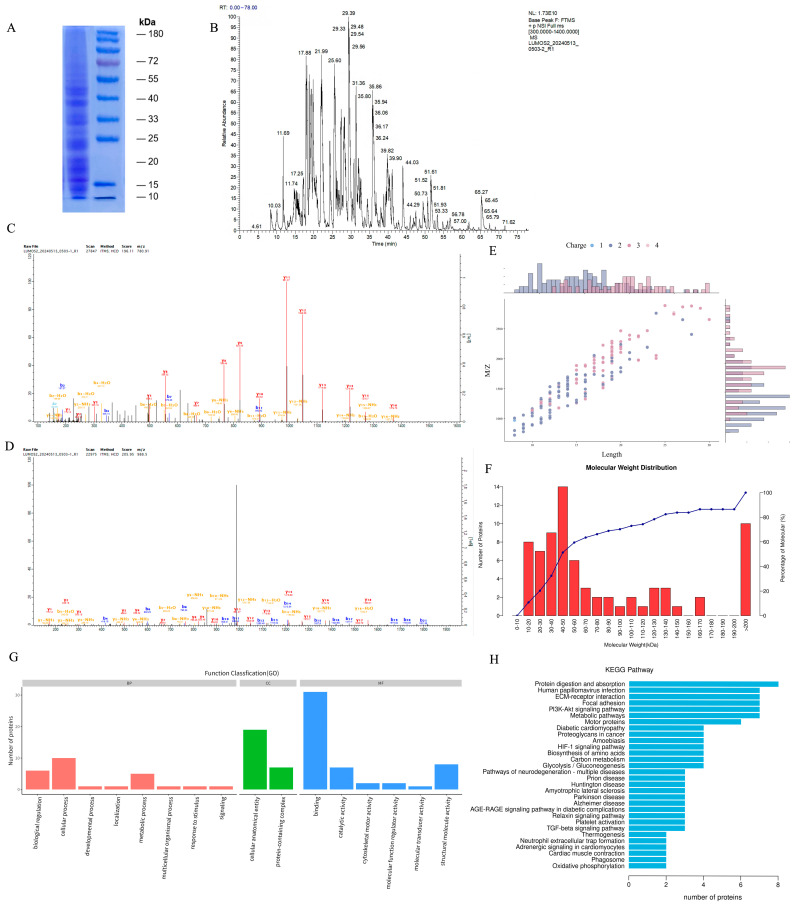
Protein identification, functional annotation, and enriched pathways of DBHE. (**A**) SDS-PAGE results for DBHE results. (**B**) Mass spectrum base peak of DBHE. (**C**,**D**) Peptides identified in DBHE. (**E**) The length of the peptide. The horizontal axis is the length of the peptide, the vertical axis is the number of the peptides, and the color indicates the charge status of the peptide. (**F**) Protein molecular weight. (**G**) Functional annotation of DBHE protein GO. (**H**) Notes on the KEGG pathway of DBHE proteins.

The SDS-PAGE results showed that DBHE has a high amount of proteins, mainly found in the molecular weight range of 15–50 kDa. A total of 69 proteins and 181 peptides were detected from DBHE using liquid chromatography–tandem mass spectrometry (LC-MS/MS), as shown in Figure 1A,B. The peptide length distribution is primarily focused within the range of 9 to 30 amino acids. The majority of the quantified protein molecular weights fall within the range of 10 to 60 kDa, accounting for 60% of the distribution (Figure 1E,F). This observation is consistent with the findings from the SDS-PAGE analysis. The 10 most abundant peptides identified by LC-MS are shown in Table 1. The intensity value is derived from the LC-MS results. The two peptides exhibiting the most intense signal in DBHE are GETGPAGPAGPIGPVGAR and SGDRGETGPAGPAGPIGPVGAR. These peptides are specifically recognized as the segments of the α1 chain of type I collagen (Figure 1C,D).

The mass spectrometry proteomics data have been deposited in the ProteomeXchange Consortium via the PRIDE partner repository with the dataset identifier PXD057258 (Appendix A).

### 3.2. Functional Annotation and Enriched Pathways of DBHE

Proteins participate in 16 biological processes: biological regulation, cellular process, developmental process, localization, metabolic process, multicellular organismal process, response to stimulus, signaling, cellular anatomical entity, protein-containing complex, binding, catalytic activity, cytoskeletal motor activity, molecular function regulator activity, molecular transducer activity, and structural molecule activity. Out of them, the three biological processes that have the greatest number of proteins are binding, the cellular anatomical entity, and the cellular process (Figure 1G).

The proteins underwent KEGG pathway annotation. The main signaling pathways associated with these proteins are protein digestion and absorption, human papillomavirus infection, ECM-receptor interaction, focal adhesion, the PI3K-Akt signaling pathway, and metabolic pathways, among others (Figure 1H).

### 3.3. Amino Acid Content

**Figure 2:**
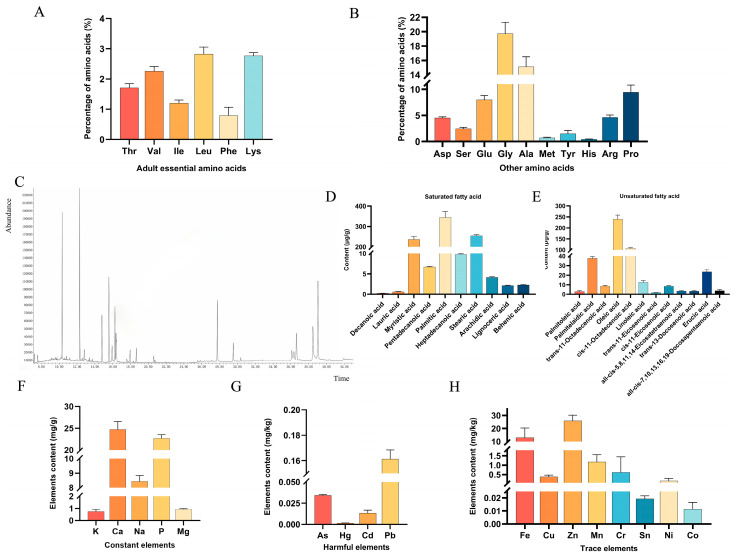
Contents of amino acids, fatty acids, and inorganic elements in DBHE. (**A**) The content of adult essential amino acids. (**B**) The content of other amino acids. (**C**) DBHE fatty acid TIC. (**D**) The content of saturated fatty acids. (**E**) The content of unsaturated fatty acids. (**F**) The content of macroelements. (**G**) The content of harmful elements. (**H**) The content of trace elements.

Among the 16 amino acids tested, the three with the highest content were glycine (18.201%), alanine (13.939%), and proline (9.770%). It can be seen from the results that DBHE not only contains a substantial amount of non-essential amino acids but also provides some essential amino acids required for adult nutrition, which is beneficial for human growth and development (Figure 2A,B).

### 3.4. Fatty Acid Content Analysis

Previous studies have shown that the GC-MS platform is suitable for non-targeted characterization of fatty acids [27]. Among the 49 free fatty acids detected, DBHE contained 22 (Figure 2C). Among these, the saturated fatty acids with the highest content in DBHE were palmitic acid (354.41 μg/g), stearic acid (254.02 μg/g), and myristic acid (233.95 μg/g). Oleic acid (240.91 μg/g) was the most prevalent unsaturated fatty acid. The trans fatty acid content of DBHE was 16.36 μg/g, which is significantly lower than the limit value of 0.3 g/100 g or 100 mL stated in the National Food Safety Standard General Rules for Nutrition Labeling of Prepackaged Foods (GB 28050-2011 [28]) for trans fatty acids (Figure 2D,E).

### 3.5. Inorganic Element Analysis

The 17 inorganic elements detected were classified according to macroelements, trace elements, and harmful elements. The highest content of DBHE is Ca and P of the macroelements (Figure 2F). DBHE contains a variety of trace elements, among which the highest content of trace elements are Zn and Fe (Figure 2H). The content of harmful elements (As, Hg, Cd, Pb) in DBHE was far below the limit standards specified in the Limit of Pollutants in Food under the National Standard for Food Safety (GB 2762-2022 [29]) (Figure 2G).

### 3.6. Cell Toxicity of DBHE

#### 3.6.1. Cell Activity Assay

**Figure 3:**
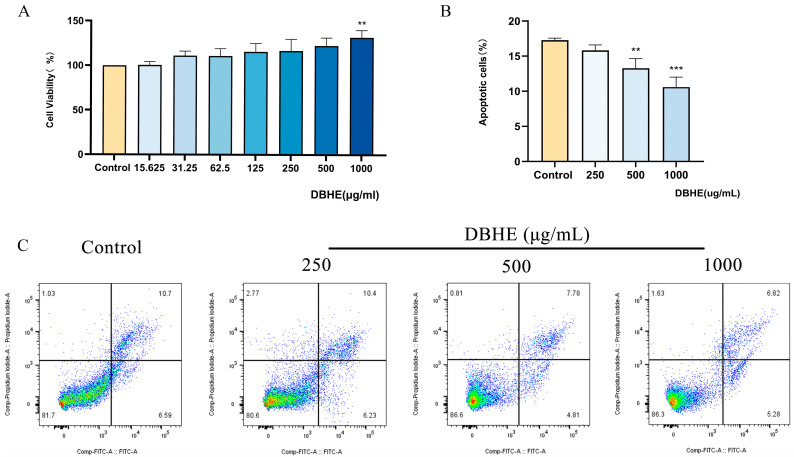
Cell toxicity of DBHE. (**A**) MC3T3-E1 cells activity assay (n = 6) (** *p* < 0.01). (**B**) Apoptosis of MC3T3-E1 cells (n = 6) (** *p* < 0.01, *** *p* < 0.001). (**C**) Flow cytometry results of different doses of DBHE.

To evaluate the potential cytotoxicity of DBHE, we analyzed its effect on MC3T3-E1 cells. Figure 3A illustrates that the administration of DBHE at moderate concentrations did not result in any detrimental effects on MC3T3-E1 cells. The growth of MC3T3-E1 cells was increased in a manner that depended on the dosage, with doses ranging from 0 to 1000 μg/mL. When the cells were treated with DBHE at a concentration of 1 mg/mL, their vitality improved to 131.83% (*p* < 0.01).

#### 3.6.2. Flow Cytometry

To systematically assess the cytotoxicity of DBHE, the apoptosis of MC3T3-E1 cells was measured by flow cytometry. For the apoptosis experiment, DBHE doses of 250, 500, and 1000 μg/mL were chosen based on the results of the CCK-8 assay. The findings indicated that the level of apoptosis in MC3T3-E1 cells decreased in a dose-dependent manner when compared to the blank control group (Figure 3B,C). There is an inverse relationship between the concentration of DBHE and the rate of apoptosis, with a statistically significant difference (*p* < 0.01).

### 3.7. Acute Toxicity of DBHE

#### 3.7.1. Effect on General Condition

**Figure 4:**
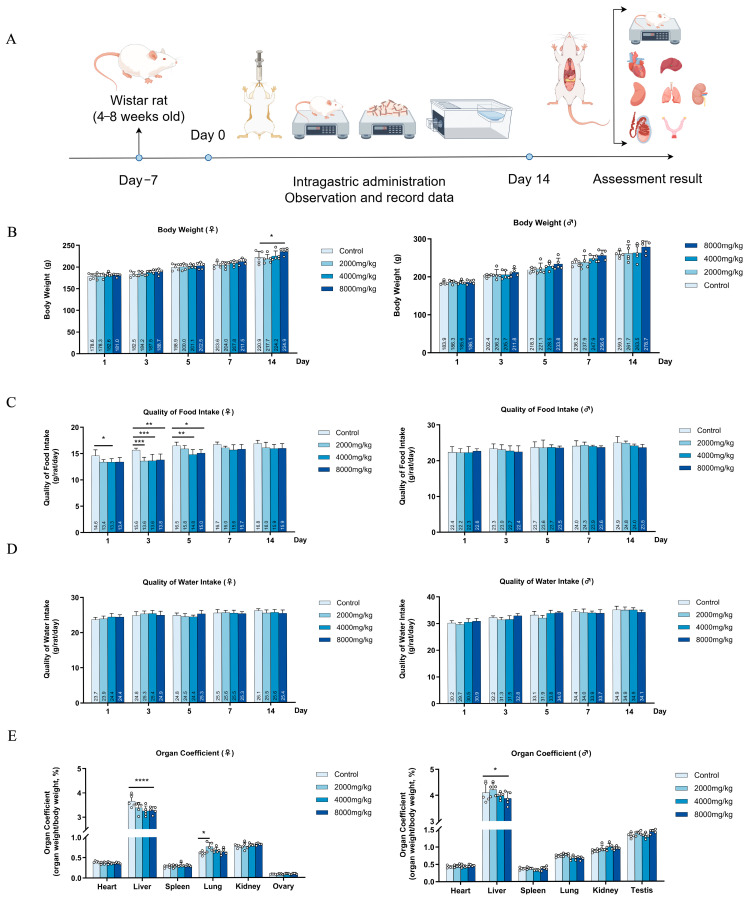
Impact of acute poisoning on rats’ body weight, food consumption, water consumption, and organ indices. (**A**) The experimental methodology for assessing acute toxicity (n = 5). (**B**) Influence on body weight. (**C**) Influence on dietary intake. (**D**) Influence on water intake. (**E**) Influence of major organs on organs. * *p* < 0.05; ** *p* < 0.01; *** *p* < 0.001; **** *p* < 0.0001.

The rats maintained their health throughout the duration of the experiment (Figure 4A). There were no discernible distinctions observed between the control group and the DBHE group in relation to pupil appearance, coat color, feces, respiration, temperature, and behavior. There was an absence of secretion in their eyes, nose, mouth, or ears. In addition, there were no aberrant situations noticed, such as seizures, lethargy, or bristling, and no deaths were recorded.

#### 3.7.2. Effects on Body Weight, Food Intake, and Water Intake

Regarding weight gain and food consumption in mice, the control group and the DBHE low-dose treatment group had similar results. It is worth mentioning that the rats in the 8000 mg/kg DBHE group had a higher body weight compared to the control group, but their food consumption was lower than that of the control group. We hypothesize that DBHE may enhance satiety and lead to weight gain in rats due to its high protein content. In terms of water intake, it remained relatively stable across all groups (Figure 4B–D).

#### 3.7.3. General Anatomical Observation

The gross pathology examination showed no pathological alterations in the tissues and organs of the treated rats. All the organs, including the heart, liver, spleen, lungs, kidneys, ovaries, and testes, were within the normal range. Nevertheless, the liver organ index in the high-dose DBHE group surpassed that of the control group, suggesting that a high intake of DBHE could potentially impact the liver function of rats (Figure 4E). However, considering variations across individuals, it is important to examine the following sub-acute toxicity tests collectively. In addition, the organ parameters of the control group did not show any significant differences compared to the DBHE group.

### 3.8. Subacute Toxicity of DBHE

#### 3.8.1. Effect on General Condition

**Figure 5:**
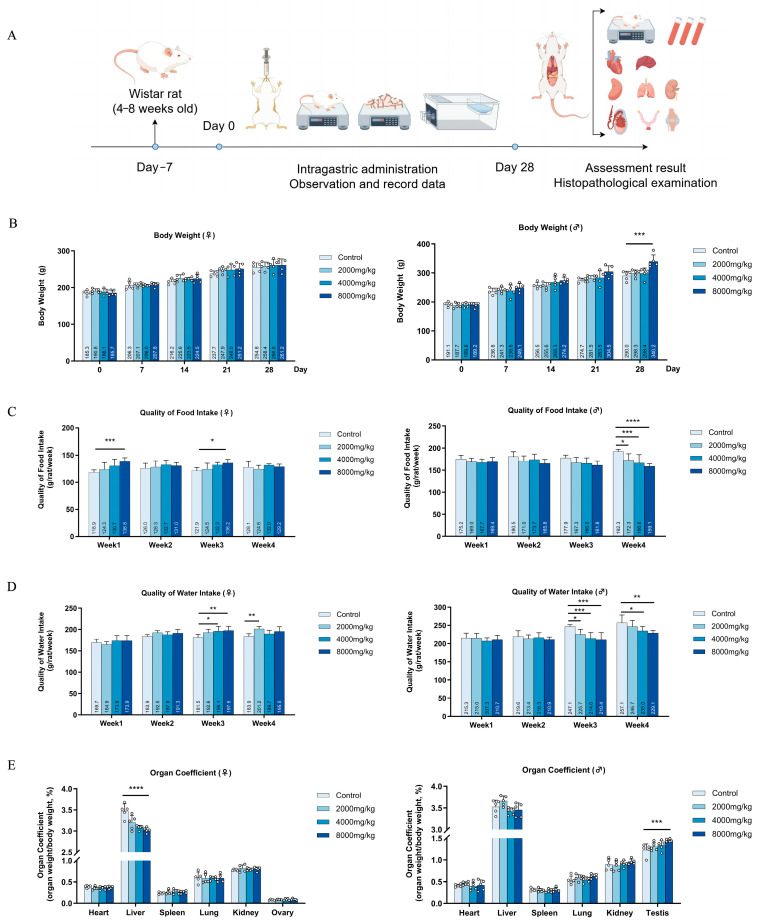
Impact of subacute toxicity on rats’ body weight, food intake, water intake, and organ index. (**A**) Influence on body weight. (**B**) Subacute toxicity test protocol (n = 5). (**C**) Influence on dietary intake. (**D**) Influence on water intake. (**E**) Influence of major organs. * *p* < 0.05; ** *p* < 0.01; *** *p* < 0.001; **** *p* < 0.0001.

The rats exhibited excellent physical condition throughout the course of the investigation (Figure 5A). The DBHE-treated group did not exhibit any anomalies in their eyes, hair color, feces, respiration, temperature, or behavior. There was an absence of secretions in his eyes, lips, and ears. During the final week of the trial, we noticed a minimal quantity of blood near the nostrils of two rats in the 8000 mg/kg DBHE group, potentially indicating internal heat. In addition, there were no aberrant occurrences like seizures, drowsiness, or raised fur, and no fatalities were recorded.

#### 3.8.2. Effects on Body Weight, Food Intake, and Water Intake

All of the rats gained weight during the experiment. Nevertheless, following the conclusion of the trial, the control group exhibited a slightly elevated body weight (Figure 5B). Furthermore, the food consumption of male rats in the experimental group was notably lower than that in the control group after 3–4 weeks. The water consumption in the treatment group was comparatively lower than that in the control group, as shown in Figure 5C,D. Hence, the prolonged administration of DBHE for a duration of 28 days resulted in specific impacts on the rats, notably the male rats, with noticeable effects on their food and water consumption, which were statistically significant (*p* < 0.05).

#### 3.8.3. General Anatomical Observation

No pathological alterations were observed in the tissues or organs of mice during gross pathology examination. Nevertheless, the liver organ index in the administration group exhibited a lower value compared to the control group, and the disparity increased as the dosage increased, with a statistically significant difference (*p* < 0.001). In addition, there was no notable distinction observed between the control group and the DBHE group in relation to other organs (Figure 5E).

During the acute toxicity experiment, the rats were administered DBHE by intragastric injection at a dose equivalent to that utilized in human clinical use. The findings revealed that the mice administered with the DBHE did not experience any notable adverse effects or harmful symptoms, therefore confirming the safety and tolerability of the dosage. Hence, the mice demonstrated a maximum tolerated dose (MTD) of DBHE that exceeded 8 g/kg, and there were no signs of toxicity upon acute administration.

#### 3.8.4. Hematology Analysis

**Figure 6:**
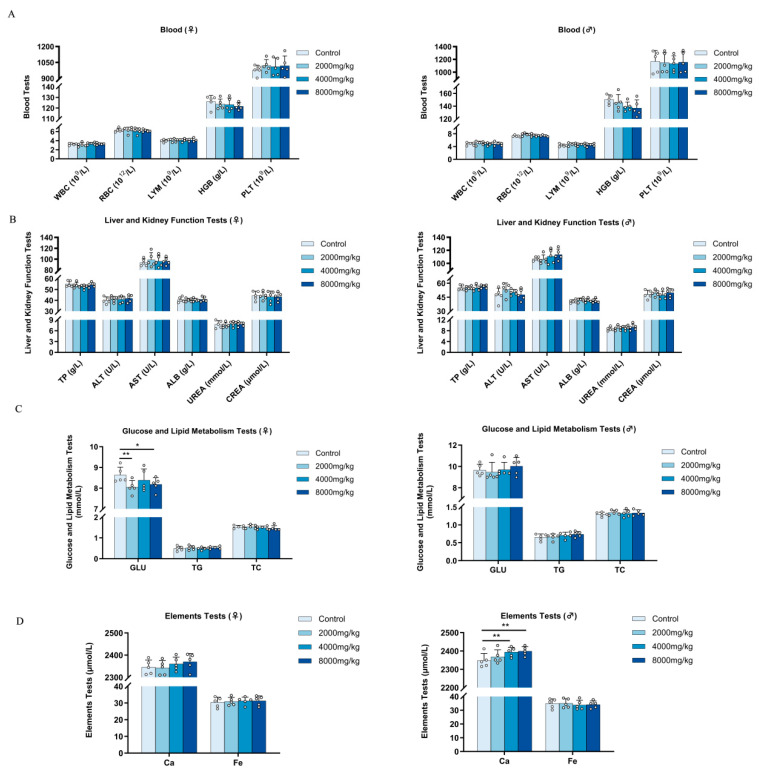
Effect of subacute toxicity on routine blood tests and biochemical markers in rats (n = 5). (**A**) Influence on body weight. (**B**) Hepatic and renal function. (**C**) Effects on the metabolism of glucose and lipids. (**D**) Influence on the elements. * *p* < 0.05; ** *p* < 0.01.

The hematology analysis showed that there was no abnormality in the treatment group. Among these findings, a slight reduction in HGB was observed in the administration group (Figure 6A). However, considering individual differences, these insignificant changes can be considered negligible. All other blood indicators were within normal ranges.

#### 3.8.5. Histopathological Examination

**Figure 7:**
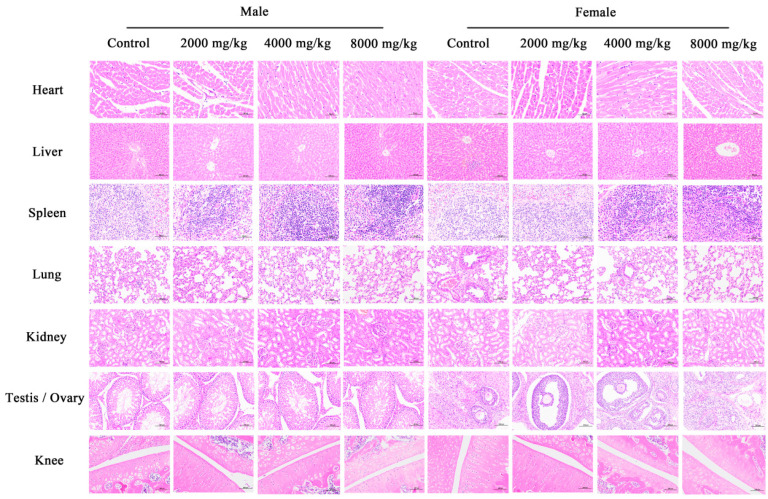
Histopathological results of eight organs in rats in subacute toxicity test. Liver, lung, kidney, testis, ovary, and knee (HE 200×); heart and spleen (HE 400×).

The histological examination (HE staining) revealed the absence of any pathological abnormalities in the cardiac, hepatic, splenic, pulmonary, renal, testicular, ovarian, and knee tissues of the rats. Inflammatory cells were occasionally observed in the field of view (Figure 7). Compared with the control group, it was found that the splenic lymphocyte distribution density was different in the DBHE treatment group, and the distribution of white pulp components in the spleen was increased, with some color differences. This suggests that the DBHE may increase the maturation process of the spleen and the number of mature lymphocytes, potentially improving lymphocyte immunity and humoral immune function [30,31], although the intergroup differences were not obvious. In addition, there were a small number of non-uniform vacuoles in the hepatic lobules in the high-dose group, which were considered to be lipid changes in hepatocytes, possibly associated with excessive protein intake and weight gain in rats. The HE staining of the knee joints showed that in the treated group, the joint surfaces were covered with hyaline cartilage, the joint capsule cavities were smooth, and there was a thin layer of synovial cells covering the surface. This indicates that there was no toxic effect on the rat joints. Other organs showed no obvious dose-dependent changes. Occasionally, phenomena such as inflammatory cell infiltration are seen in the visual field, and these changes may be due to differences in the eating environment and the individual, but they are not considered to be pathologic changes caused by DBHE ingestion.

## 4. Discussion

As a relatively understudied raw material for health foods, conducting component analysis and toxicity evaluations of deer bone is of great significance. Modern studies have shown that the water extract of deer bone can prevent memory impairment induced by scopolamine in mice [32], alleviate symptoms of knee osteoarthritis [33,34,35], and mitigate neutropenia and other diseases [36]. However, due to its unclear material basis and the lack of systematic safety evaluations, deer bone has not yet been widely used in health foods.

Our research results indicate that through LC-MS/MS analysis [37], the deer bone hydroethanolic extracts contain 69 proteins and 181 peptides. The peptides are predominantly found within the range of 7 to 30 amino acids in length. The majority of peptides originate from the α1 chain of type I collagen, indicating that the primary outcome of DBHE is the breakdown of type I collagen. These peptides have the ability to influence several processes, including protein digestion and absorption, human papillomavirus infection, and ECM-receptor interaction. On this basis, DBHE holds promise for the development of various functional foods, skincare products, and biomedical products [38,39]. Amino acid analysis showed that among the 16 amino acids detected, the highest content was glycine, alanine, and proline. Existing studies have shown that gas chromatography quantitative analyses of amino acids in skin and bones both display the molecular distribution characteristics of collagen, mainly consisting of glycine and small amounts of proline, hydroxyproline, and alanine [40]. Therefore, based on the amino acid composition characteristics of deer bone, it can be concluded that deer bone contains a large amount of collagen. The various bioactive peptides obtained from the hydrolysis of collagen have been widely applied in health foods, skincare products, and biomedical product-related fields [41,42,43]. In the determination of 49 free fatty acids, we found that DBHE contains 22 fatty acids. Among them, the most abundant fatty acids are palmitic acid, stearic acid, and oleic acid. Interestingly, in studies of modern deer-derived products, deer antlers, and venison also contain relatively high levels of saturated fatty acids such as palmitic acid, stearic acid, and oleic acid [44,45]. This result suggests, to some extent, that the fatty acid composition of deer-derived products is relatively stable, and there may be synergistic effects in the functions related to these fatty acids. ICP-MS results showed that the elements present in the highest concentrations in DBHE were the macronutrients Ca and P. Additionally, DBHE contains multiple trace elements, with Zn and Fe being the most abundant. Żaneta et al. analyzed 21 mineral elements in the bones of farmed fallow deer and found that the highest contents in the deer bones were Ca, P, and Ba [46]. Therefore, Ca and P play a crucial role in growth and development, and our extracted DBHE not only retains these elements but also provides a variety of trace elements.

In order to guarantee the safety of DBHE as a functional food, we undertook a thorough investigation of its components and carried out toxicity experiments both in vitro and in vivo. Viability and apoptosis studies were conducted on MC3T3-E1 cells at various dosages. It was shown that DBHE did not display any harmful effects within the dose range of 1 mg/mL. In fact, it stimulated the proliferation of MC3T3-E1 cells in a manner that was dependent on the dose. The flow cytometry results demonstrated that DBHE attenuated the apoptosis of MC3T3-E1 cells. The findings indicate that DBHE is not harmful to cells and has a specific capacity to enhance the proliferation of osteoblasts. This provides important information for considering the prospective application of deer bone in the treatment of bone disorders such as osteoporosis [47,48].

We conducted acute and subacute toxicity studies on animals by orally administering DBHE at various doses to rats. Throughout the treatment period, the rats maintained fair physical condition, and no notable histological alterations were detected. The data indicate that the treatment group had a decreased food intake compared to the control group, suggesting that DBHE, with its high protein content, induces a feeling of fullness in the rats [49]. In addition, routine blood tests or assessments of kidney function, sugar–lipid metabolism, and ion metabolism did not reveal any obvious toxic effects. Overall, after four weeks of administration, DBHE did not exhibit any toxic effects at the doses we tested. Furthermore, by observing histological sections of tissues from animals, the degree and type of tissue damage can be assessed, which helps in determining the toxicity and safety of the DBHE [50]. In the subacute toxicity study, through histological examination, we observed that the liver morphology was normal compared to the control group. However, in the high-dose treatment group, there were a few uneven vacuoles in the hepatic lobules, suggesting possible lipid changes in the hepatocytes, which may be related to excessive protein intake and weight gain in the rats. Other organs occasionally showed minor inflammatory cell infiltration, but considering individual variability and other factors, this was not considered to be pathological damage caused by DBHE. The organs had normal morphology and function. This suggests that DBHE did not exhibit any toxic effects at the doses we tested.

This study not only provides a new method for the extraction of deer bone but also uses label-free proteomics technology to perform quantitative analysis of the main components of DBHE. It systematically evaluates its safety both in vitro and in vivo and, through its promoting effect on the proliferation of MC3T3-E1 cells, reveals the potential anti-osteoporosis and anti-arthritis effects of DBP. This study provides dependable facts to encourage the advancement and utilization of health foods derived from deer bones.

## 5. Conclusions

DBHE was obtained through reflux extraction after being mixed with water and ethanol. Using multilevel mass spectrometry, we quantitatively analyzed 69 proteins, 181 peptides, and 16 amino acids in DBHE, as well as 22 fatty acids and 17 inorganic elements. It can be concluded that DBHE has a rich protein content and can provide a variety of essential amino acids for adults. Meanwhile, trans fatty acid and harmful element contents are far below relevant food standards. Then, we conducted cell experiments to verify that DBHE was non-toxic in both cell proliferation and apoptosis assays. We find that within a dose range of 1 mg/mL, DBHE can promote proliferation and reduce apoptosis in MC3T3-E1 cells. In acute and subacute toxicity experiments, all measurements remain within acceptable thresholds, and Wistar rats are not at risk. This suggests that DBHE did not exhibit any toxic effects at the doses we tested and also supports the potential of the extract to be used in the development of health foods.

## Figures and Tables

**Table 1:** DBHE identified the top 10 most abundant peptides by LC-MS.

Sequence	Score	Mass (Da)	Intensity DBHE	Protein Names	Accession
GETGPAGPAGPIGPVGAR	169.65	1559.8056	1.07 × 10^11^	collagen alpha-1(I) chain isoform X4	XP_043327121.1
SGDRGETGPAGPAGPIGPVGAR	220.72	1974.9872	8.03 × 10^10^	collagen alpha-1(I) chain isoform X4	XP_043327121.1
PGPIGPAGAR	138.24	891.49265	2.30 × 10^10^	collagen alpha-2(I) chain isoform X1	XP_043318553.1
GELGPVGNPGPAGPAGPR	173.76	1598.8165	1.61 × 10^10^	collagen alpha-2(I) chain isoform X1	XP_043318553.1
GEAGPAGPAGPAGPR	206.73	1260.6211	1.45 × 10^10^	collagen alpha-2(I) chain isoform X1	XP_043318553.1
DGEAGAQGPPGPAGPAGER	211.22	1689.7707	1.29 × 10^10^	collagen alpha-1(I) chain isoform X4	XP_043327121.1
STGISVPGPMGPSGPR	139.76	1495.7453	1.11 × 10^10^	collagen alpha-1(I) chain isoform X4	XP_043327121.1
VADIGIGISGQEGMQ	141.91	1473.7133	6.31 × 10^9^	phospholipid-transporting ATPase VD isoform X2	XP_043294276.1
RGETGPAGPAGPIGPVGAR	149.6	1715.9067	6.14 × 10^9^	collagen alpha-1(I) chain isoform X4	XP_043327121.1
GPSGPQGIR	153.73	867.45626	4.71 × 10^9^	collagen alpha-2(I) chain isoform X1	XP_043318553.1

## Data Availability

The original contributions presented in this study are included in the article; further inquiries can be directed to the corresponding author.

## Appendix A

The mass spectrometry proteomics data have been deposited in the ProteomeXchange Consortium via the PRIDE partner repository with the dataset identifier PXD057258.
